# Giant Perineal Solitary Fibrous Tumor: A Rare Case Report

**DOI:** 10.1155/2017/4876494

**Published:** 2017-03-02

**Authors:** Petronio Augusto de Souza Melo, Ana Maria Yoshino Bonifaci, Fabio da Silva Crochik, Claudio Bovolenta Murta, Joaquim Francisco de Almeida Claro, Joao Padua Manzano

**Affiliations:** ^1^Division of Urology, Men's Health Centre, Hospital Brigadeiro, Sao Paulo, SP, Brazil; ^2^Division of Pathology, Men's Health Centre, Hospital Brigadeiro, Sao Paulo, SP, Brazil; ^3^Federal University of Sao Paulo, Sao Paulo, SP, Brazil

## Abstract

*Background*. Solitary fibrous tumor (SFT) is a fibroblastic mesenchymal tumor that was initially described from the pleura but currently arises at almost every anatomic site. It is usually benign, and surgical resection is curative. SFT involving the perineum is extremely rare. This is the third case report of a perineal SFT in the literature.* Case Presentation*. We reported an uncommon case of a 64-year-old man presenting with a huge perineal mass that started growing 3 years before his arrival in our service. He was asymptomatic. A contrast-enhanced CT scan revealed a heterogeneous well-circumscribed perineal mass with soft-tissue density. Invasion of the surrounding organs, distal metastasis, and lymph node swelling were absent. The complete resection of mass was done successfully. The specimen was a 23.0 × 14.0 × 8.0 cm encapsulated tumor. Mass weight was 1,170 g. After pathological analysis, we confirmed that the mass was a solitary fibrous tumor. The diagnosis was based on clinical findings and histological morphology and immunohistochemistry study.* Conclusion*. SFTs are usually indolent tumors with a favorable prognosis. The perineal location is extremely rare. Complete resection of the mass is the treatment of choice.

## 1. Introduction

Solitary fibrous tumor (SFT) is a rare fibroblastic mesenchymal tumor initially described in the pleura and lately documented at almost every anatomic site and organ [1], including soft tissue and viscera, albeit with a peculiar predilection for body cavity sites, including pleura, peritoneum, and meninges.

SFTs accounted for less than 2 percent of all soft-tissue tumors cases presenting to a large referral center in one series, demonstrating how rare this neoplasm is [2]. SFTs may arise at any age but are most common in the fifth to seventh decades. Men and women are affected with equal frequency. There are no known predisposing risk factors.

About 30 percent of SFTs arise in the peritoneal cavity, retroperitoneal soft tissue, or pelvis (including visceral sites) and they constitute the largest site-related group in most series of extrapleural SFTs [3]. The most common intra-abdominal site is the retroperitoneum, followed by the pelvic soft tissues.

The most common presentation of soft-tissue SFT is a painless mass [3, 4]. If the tumor impinges upon a nerve, paresthesias or other nerve symptoms may occur. SFTs are frequently slow-growing and the mass can enlarge over several years. Soft-tissue SFTs tend to be smaller at presentation than pleural or intra-abdominal SFTs, likely due to the relative ease of detection earlier in the course of disease.

There are few cases of this tumor arising in perineum reported in the literature. We present here a case of a large perineal mass revealed to represent a SFT.

## 2. Case Report

### 2.1. Clinical Findings

**Figure 1:**
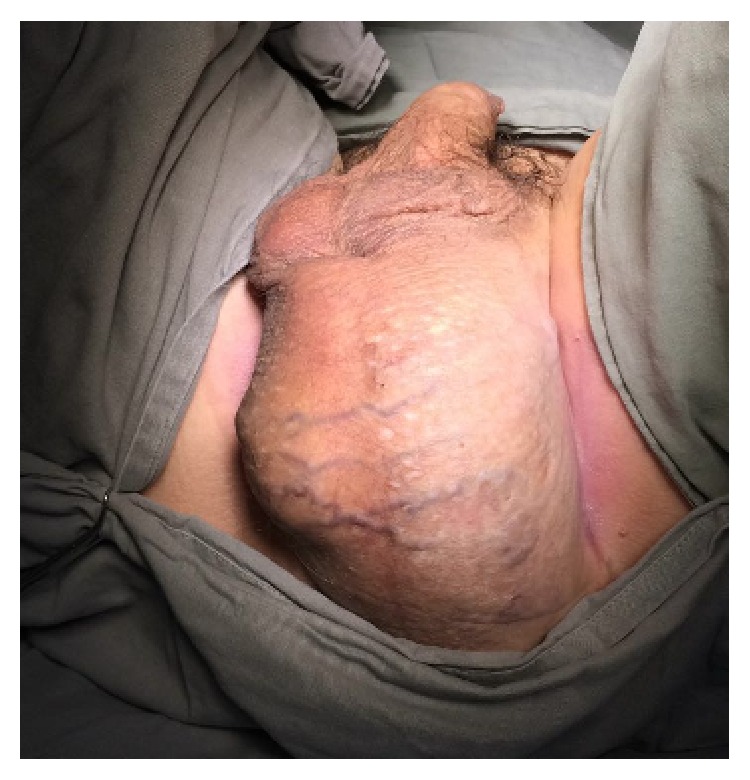
Perineal mass prior to surgery.

A 64-year-old man presented in our urological service complaining of a huge mass in his perineum. He described a slow-growing, painless mass, and he noticed it the first time 3 years ago (Figure 1). He had no urinary or gastrointestinal symptoms. In his medical past, he had arterial hypertension and a previous myocardium infarction. He was a former smoker (40 pack-years) and had no surgical previous history. In the physical examination, there was a large perineum mass of about 20 cm, with a uniform and smooth consistency, apparently without involvement of the testis or urethra. The rectal mucosa was normal in the digital rectal examination.

### 2.2. Image Diagnosis

**Figure 2:**
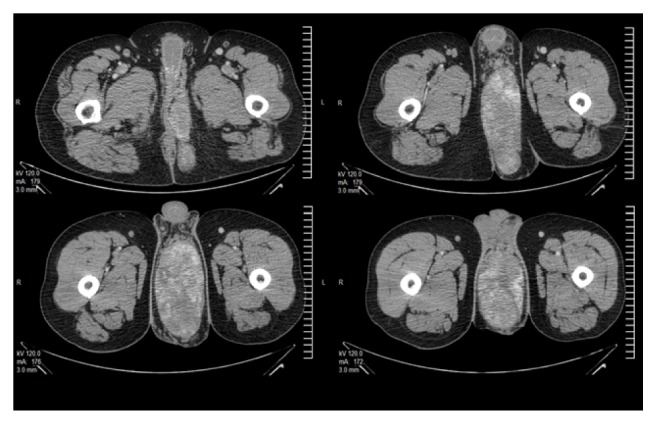
CT scan showing a large smoothly marginated perineal mass with uniform soft-tissue attenuation.

The CT scan showed a bulky, lobulated, hypervascularized, and heterogenous mass in soft tissue of the perineum measuring 18.0 × 12.0 × 7.7 cm (860 cc) (Figure 2). CT of thorax, abdomen, and pelvis showed no other abnormalities.

### 2.3. Surgical Treatment

**Figure 3:**
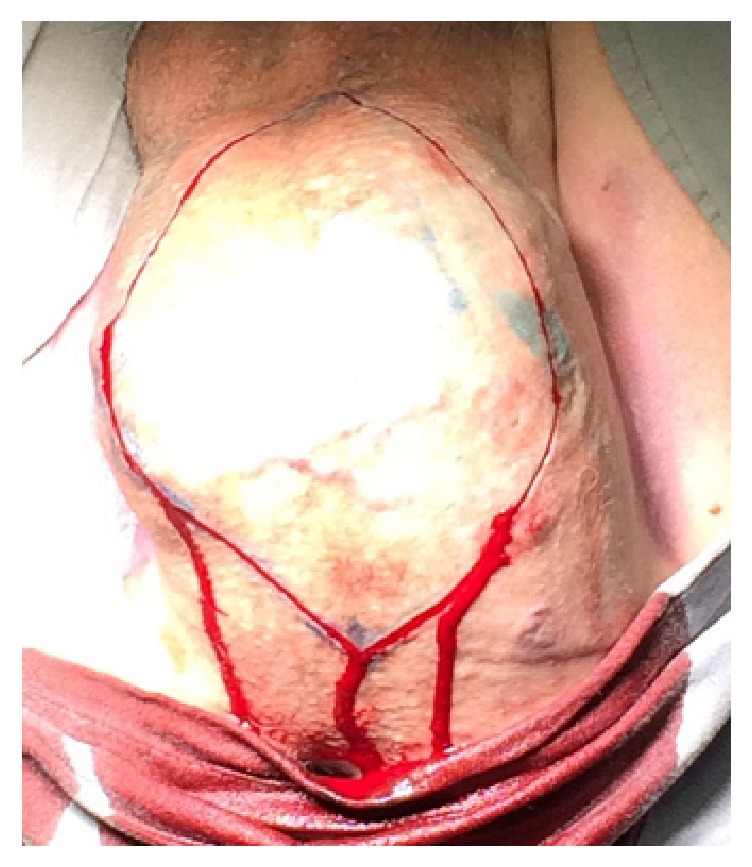
Surgical incision.

**Figure 4:**
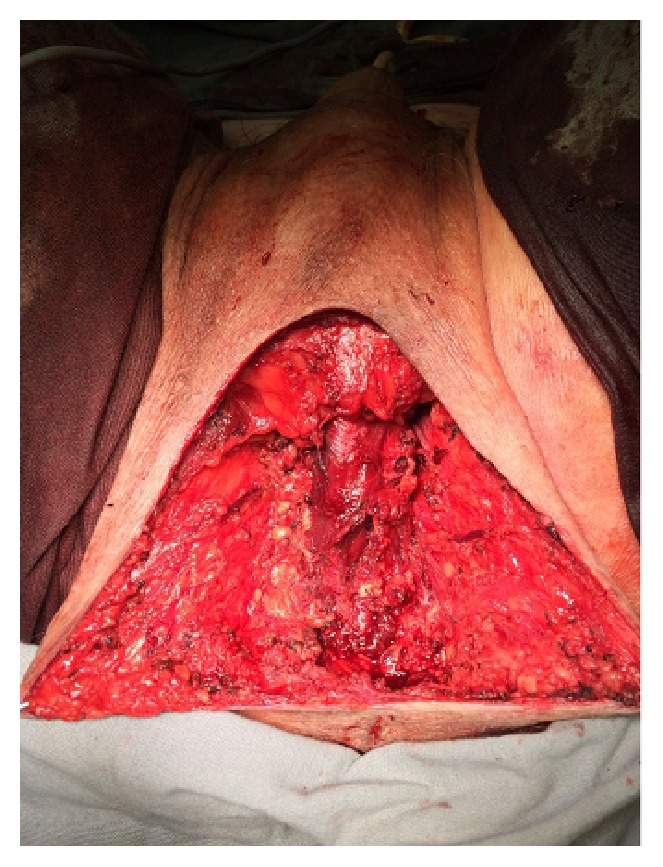
Perineum after mass resection.

**Figure 5:**
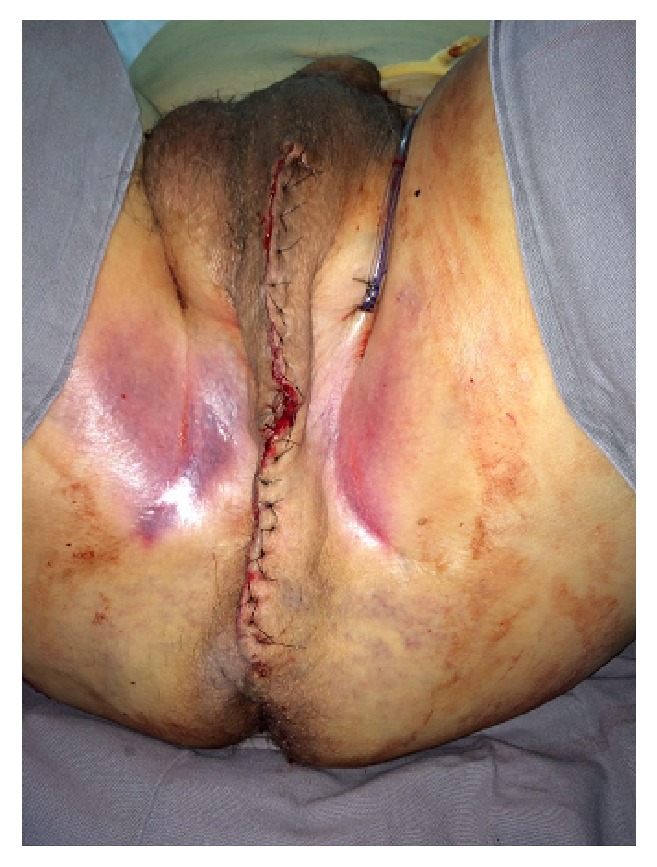
Perineum after mass resection.

**Figure 6:**
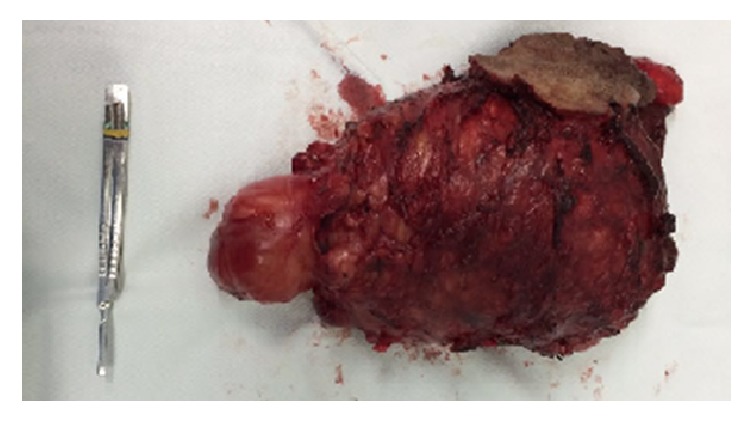
Surgical specimen.

**Figure 7:**
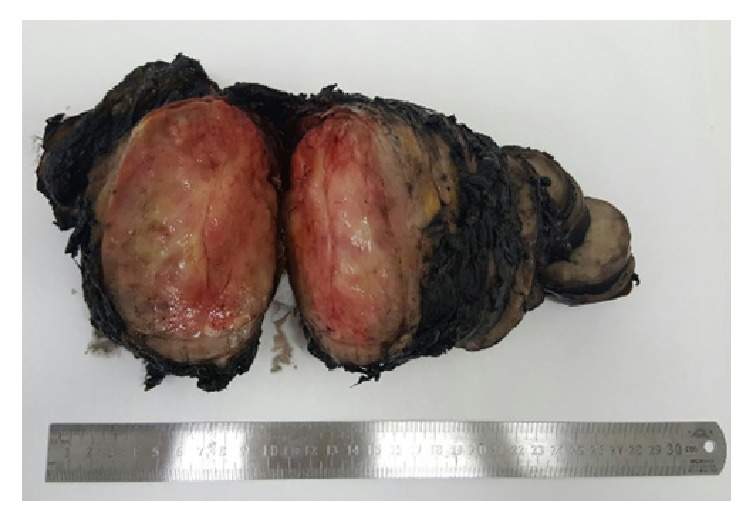
Surgical specimen.

He was submitted to excision of the mass (Figures 3–5). The tumor did not compromise the urethra, rectum, or adjacent organs and was excised completely without any complications. We used a perineal midline incision to approach the mass. A nodular structure has been resected measuring 23.0 × 14.0 × 8.0 cm and weighting 1,170 g (Figures 6 and 7).

### 2.4. Postoperative Period

After the surgery, the patient was sent to ICU prophylactically due to his medical past of myocardial infarction, where he remained for only one day. No blood transfusion was necessary. The patient presented no complaints during the length of stay. No kind of peri- or postoperative complication occurred. He had a length of hospital stay of 4 days.

### 2.5. Pathological Analysis

Macroscopic examination of the tumor demonstrated a 23.0 × 14.0 × 8.0 cm, well-defined, firm mass without necrosis and with a pale cut surface.

Pathological findings were of a solitary fibrous tumor which had variable cellularity, moderate to high, with a morphological pattern predominantly fusocellular without formation of bundles, in a haemangiopericytic arrangement, with sclerotic stroma. Microscopically, the mass was completely encapsulated. The neoplasm was composed of small, tightly packed, ovoid to spindled cells and ill-defined eosinophilic cytoplasm. Tumor cell nuclei showed open chromatin, giving a vesicular appearance. The tumor cells were arranged in a haphazard pattern around thin walled ramifying blood vessels in a fibrous stroma. There were mastocytes along the tumor. In some areas, the tumor showed atypia and pleomorphism. Mitotic activity was 1-2 mitosis per 10 HPF. Necrosis was not present.

### 2.6. Immunohistochemical Analysis

**Figure 8:**
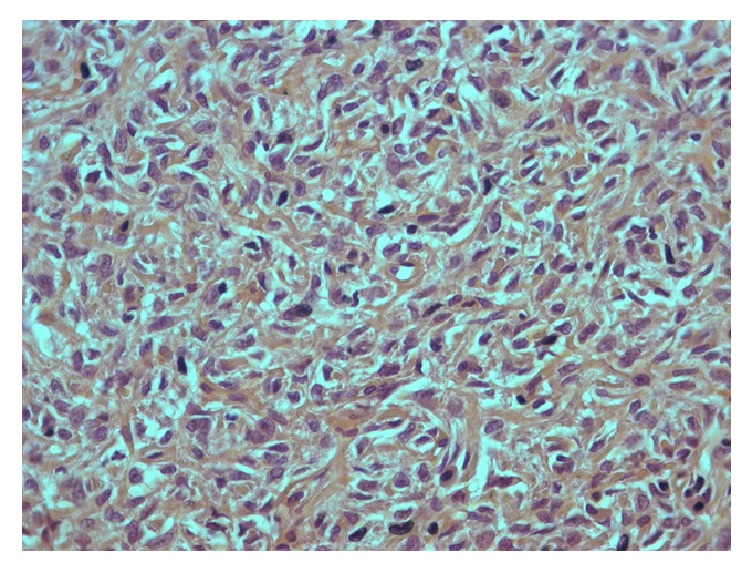
Hematoxylin and eosin-stained section.

**Figure 9:**
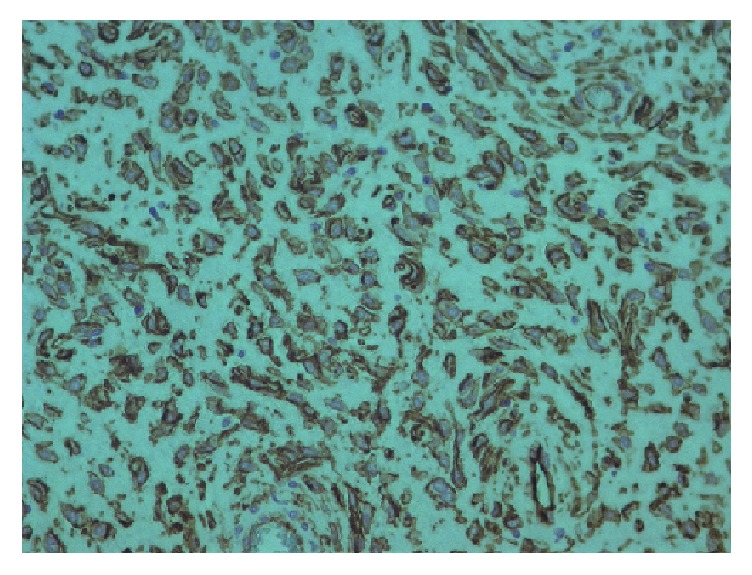
Immunohistochemistry CD34.

**Figure 10:**
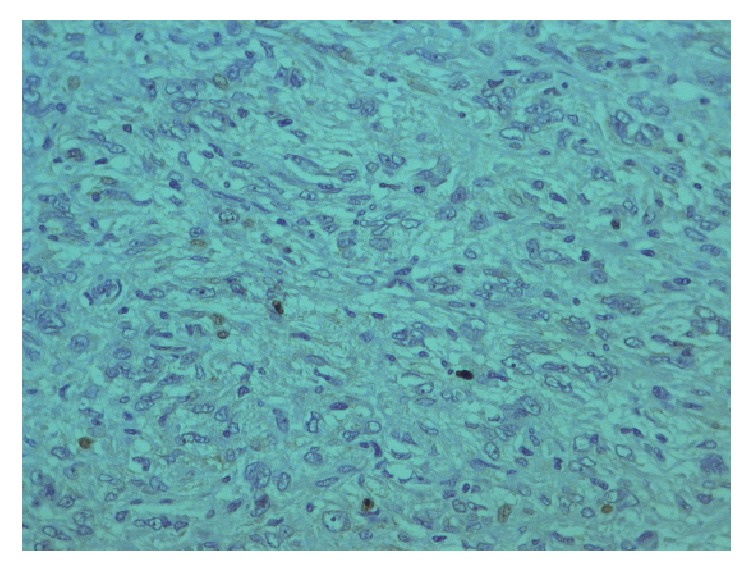
Immunohistochemistry Ki67.

**Figure 11:**
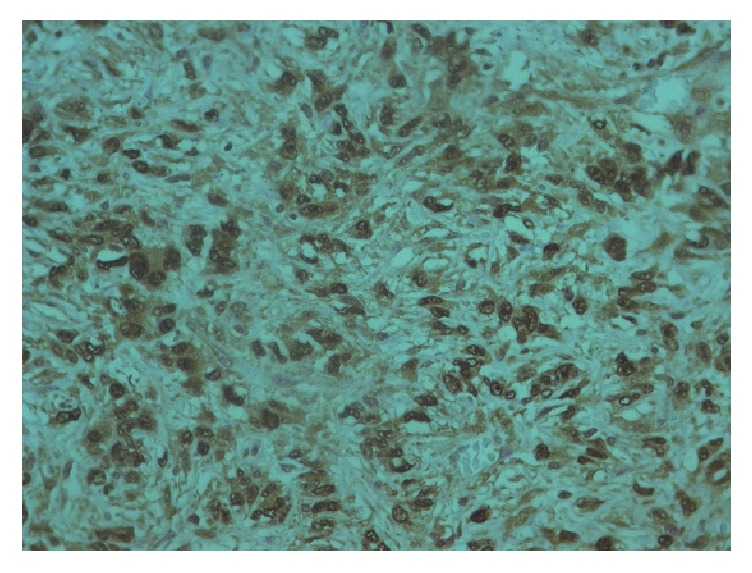
Immunohistochemistry STAT6.

The tumor showed strong and diffuse staining for CD34, CD99, bcl-2, and STAT- 6; other markers, such as pan-cytokeratins, desmin, alpha-smooth muscle actin, DOG-1, C-Kit, Glut-1, and S-100 protein, were negative and had a low Ki67 (positive in 15% of neoplastic cells). EMA was focally positive. Based on morphological and immunohistochemical findings, a diagnosis of solitary fibrous tumor was determined (Figures 8–11).

### 2.7. Follow-Up

**Figure 12:**
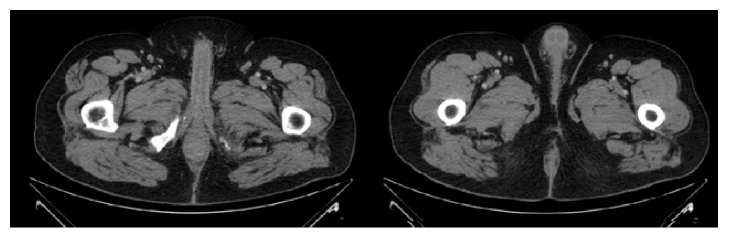
CT scan done 1 year after resection: no evidence of recurrence.

**Figure 13:**
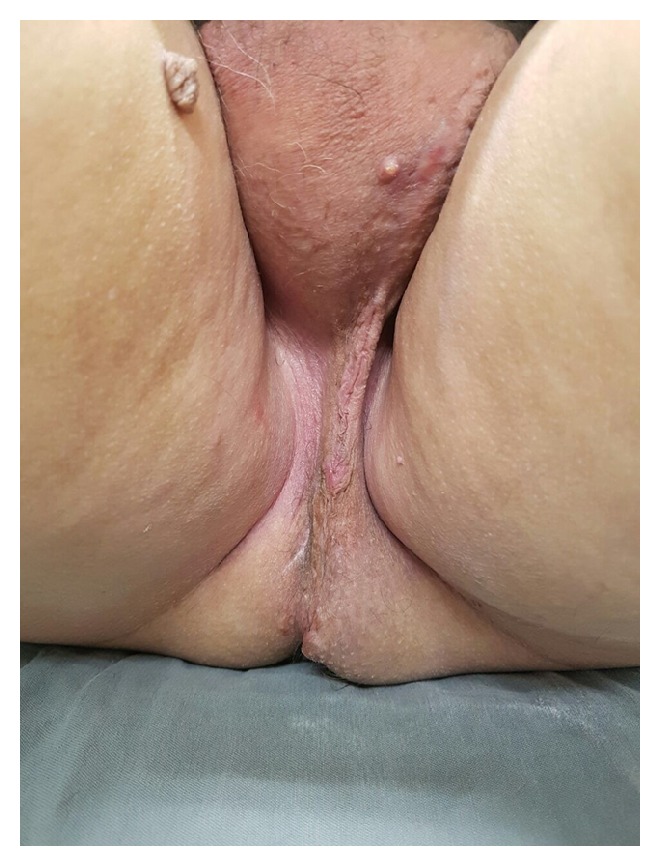
Perineal appearance after one year of tumor resection.

We have one year of follow-up after surgery. He was evaluated in outpatient setting and he was very satisfied, without any complaints. A control CT scan was done one year after resection showing no recurrence (Figure 12). The external appearance of perineum was excellent (Figure 13).

## 3. Discussion

SFTs are rare mesenchymal spindle cell neoplasms and important differential diagnosis in all anatomical locations. The first cases of primary SFT were published by Klemperer and Rabin in 1931 [5]. They have a benign nature, but it can also be associated with malignancy in rare situations [6].

Perineal location is extremely rare. The case described here is the third one reported in the literature. The first one was described by Suster et al. [7] in a case series of 12 cases of soft-tissue tumors. Kim et al. [8] described the second one in 2009 in his case report of a fat-forming solitary fibrous tumor involving the perineum.

Lesions tend to present as slowly growing mass or nodule as we could see in our case, or it can present with symptoms due to mass or pressure effects on adjacent structures. Intra-abdominal SFTs can present as a palpable mass, accompanied by pain and weight loss. Sometimes, SFTs occur in urinary tract and patients may complain of urinary symptoms including dysuria, urinary retention, hematuria, and nocturia motivating them to go to a urological clinic [9]. Gastrointestinal symptoms such as constipation, incontinence, or vomiting have also been reported. Intra-abdominal SFTs may attain large sizes (>20 cm) prior to presentation, while soft-tissue SFTs tend to be smaller likely due to the relative ease of detection earlier in the course of disease.

Rarely, SFTs can develop paraneoplastic syndromes, most commonly hypoglycemia. It can happen with SFTs arising in all sites. Hypoglycemia can occur due to tumor secretion of large insulin-like growth factor II (IGF2) [10].

CT or MRI can help to identify SFTs and often reveals proliferation of fibrous tissues as well as tumor and adjacent tissue details to facilitate decisions for surgical tumor removal. Radiologically, SFTs are often large, well-defined, lobulated, solid, and vascular masses, often with prominent feeding vessels or sometimes a visible fatty component, which displace adjacent structures [11]. Radiologic features correlating better with malignancy have included tumor size, heterogeneous signal intensity, and heterogeneous contrast uptake on magnetic resonance imaging [12]. However, the findings are similar to those of other soft-tissue tumors. No pathognomonic features are specific.

In our case, bleeding during surgery was unremarkable; no red blood cells transfusion was necessary; but there are some cases reported in the literature of massive hemorrhage during surgical resection due to the hypervascular nature of SFTs [13].

Complete resection is required for full histopathologic evaluation. Fine needle aspiration biopsies and core biopsies are inadequate. Diagnosis is based upon recognition of typical morphologic features in conjunction with a characteristic immunophenotype.

Grossly, tumors are well circumscribed, with a fibrous pseudocapsule or serosal lining. Large tumors may present hemorrhage, necrosis, or calcification [14]. In the pelvis, other tumors that can mimic SFTs include mesothelioma, ovarian Brenner tumor, and fibroma or fibrothecoma, as well as uterine leiomyoma.

Immunohistochemical analysis is paramount during differential diagnosis with other soft-tissue tumors. Markers such as CD34, CD99, BCL2, and LSD1 are commonly applied, although they are not sufficiently sensitive or specific. But new markers such as STAT6, used in our case, are more accurate in the diagnosis of SFT. Ouladan et al. [15] showed in a study of 80 SFTs that STAT6 staining was expressed in all cases; in contrast, only 1% of non-SFT mesenchymal tumors showed a nuclear STAT6 staining pattern. However, STAT6 positivity alone may not be sufficient to distinguish some cases of SFT from its histologic mimic well-differentiated/dedifferentiated liposarcoma, as these tumors also rarely overexpress full-length STAT6 [16].

Again, the majority of SFTs have an indolent course with low risk of local recurrence or metastasis. A minority of tumors recur locally or metastasize to distant sites. Some histologic features have been used to attempt to discover malignant SFTs: high mitotic activity; presence of necrosis or hemorrhage; increased cellularity; nuclear pleomorphism; stromal infiltration beyond pseudocapsule or vascular invasion.

Complete* en bloc* surgical resection to negative margins is the mainstay of therapy for all localized SFTs [17]. R0 resection is always pursued, given the low overall metastatic potential and the lack of effective adjuvant therapy.

There is no consensus about SFTs follow-up, but continued long-term surveillance is advised because of the indolent natural history and possibility of late recurrence up to 20 years after initial treatment [18].

## 4. Conclusion

Solitary fibrous tumors have usually an indolent course with a favorable prognosis. The perineal location is extremely rare. Complete resection of the mass is the treatment of choice and it is associated with high rates of success and low recurrence.
